# How to make more people adopt healthy behaviors? Assessing health literacy, health promoting lifestyle and their association of community residents in Shenzhen, China

**DOI:** 10.3389/fpubh.2022.900883

**Published:** 2022-08-15

**Authors:** Lei Zhang, Jia Liao, Xueyan Pan, Dongmei Liang, Jinmei Zeng, Mingwei Sun, Xiaowen Luo, Xingyu Ma, Mingjuan Yin, Jingdong Ni

**Affiliations:** Precision Key Laboratory of Public Health, School of Public Health and Institute of Public Health and Wellness, Guangdong Medical University, Dongguan, China

**Keywords:** health literacy, health-promoting lifestyle, reward and punishment analysis, Shenzhen, online survey

## Abstract

**Introduction:**

Health literacy (HL) has been concerned a key factor for determining the use of health information and promoting health. The study aimed to explore the relationship between different health literacy types and health promoting lifestyle (HPL) in different health literacy population.

**Methods:**

The survey analyzed a sample of 16,921 community residents in Shenzhen. The Chinese Citizen Health Literacy Questionnaire and health-promoting lifestyle profile II (HPLP- II) were used to assess health literacy and health promoting lifestyle.

**Results:**

Participants were divided into different populations based on the correlation between HL and HPL. The low-HL and medium-HL populations were judged to lack health literacy, and demographic characteristics were significantly different between different HPL levels in low-HL and medium-HL populations. There were 6 types of HL, and health information literacy (β = 0.08, *P* < 0.001) and chronic disease literacy (β = 0.08, *P* < 0.001) positively predicted HPL in the low-HL population. In the medium-HL population, the results of reward and punishment analysis showed that health information was a basic factor, chronic disease was performance factor, medical care was a motivating factor for HPL; there were 6 dimensions of HPL, and health responsibility (HR), stress management (SM) and physical activity (PA) were not significantly different in medium-HL population. The results of regression analysis showed that HR and PA had a great impact on HPL (HR: β = 0.193, PA: β =0.179, β for other dimensions was 0.186, 0.176, 0.171, 0.164), but the HR and PA standardized scores were lowest in the HPL dimensions (HR: 69.42, PA: 68.5, lower than other dimensions), so it may be HR and PA that cause HPL unchanged between groups in the medium-HL population.

**Conclusions:**

Different HL levels have different relationships with HPL, and different HL types have different effects on HPL. Shenzhen community residents need to improve their HL, and they have great potentials for further progress to improve the population health. Public health policy makers need to consider formulating different policies for people with different HL levels.

## Introduction

Health literacy (HL) is an important part of citizen health diathesis, which refers to the ability and basic diathesis of individuals to obtain health information through various channels, correctly understand and use this information, and make health-related decisions to achieve maintenance or improvement of their quality of life (1). HL plays an important role in infectious diseases (2), and it is also seen as a crucial tool for the prevention of non-communicable diseases (3) as it can reduce the possibility of indulging in health-damaging behavior (4) and has a positive impact on the health behaviors in the general population (5). As early as 1974, Simonds (6) had pointed out that improving HL should be an important component of public social policy. Studies from multiple countries have shown that low HL negatively affects health, health-related and illness-related behavior, and the utilization of health care resources (7–11). Currently, HL is still low in many countries. A HL survey in the German population showed that 54.3% of participants were found to have limited health literacy (12). The European health survey found that almost half of adults in eight countries had inadequate or problematic health literacy (13). Tehrani Bani Hashemi et al. examined HL in 15 provinces of Iran and found out that health literacy in Iran is low (14). According to the Turkey Health Literacy Survey (2014), 64.6% of the general adult population have inadequate or problematic HL levels (15). A systematic review on the prevalence of limited HL in Southeast Asia (ie, Laos, Malaysia, Myanmar, Singapore and Thailand) has reported that over 50% of the population showed limited HL (16). And in China, according to the results of national HL monitoring in 2012 to 2021 (8.8, 9.5, 9.8, 10.3, 11.6, 14.2, 17.06, 19.2, 23.2, and 25.4%, respectively) (17), the HL of Chinese residents was still at a low level.

Health-promoting lifestyle (HPL) is a healthy lifestyle. It is a spontaneous and multi-level behavior and perception that individuals engage in to maintain or improve their health level in order to achieve self-satisfaction and spiritual growth, including health-protective behavior and health-promoting behavior (18). A survey in Saudi university students showed that HPL among university students in Saudi Arabia are limited, where the majority of them have unhealthy eating habits and poor physical activity level (19). Study in Turkey adolescents also showed that HPL was at a medium level (20). In Shandong Province, China, 54.50% of adults had poor or average HPL (21). And the mean score for Taiwan adolescent HPL was 60.76 (± 11.88), which was considered moderate (22). HPL is a model for individuals to take control of, maintain, and/or enhance their own health (23) and an important determinant of health status (19). HPL can indeed free one from diseases (24), individuals following a health promoting lifestyles were healthier and suffered less from the pains of diseases (25, 26). The higher the HPL score was, the higher the quality of life (23). So that people can adopt HPL to reduce the incidence of diseases and stay healthy (27).

**Figure 1:**
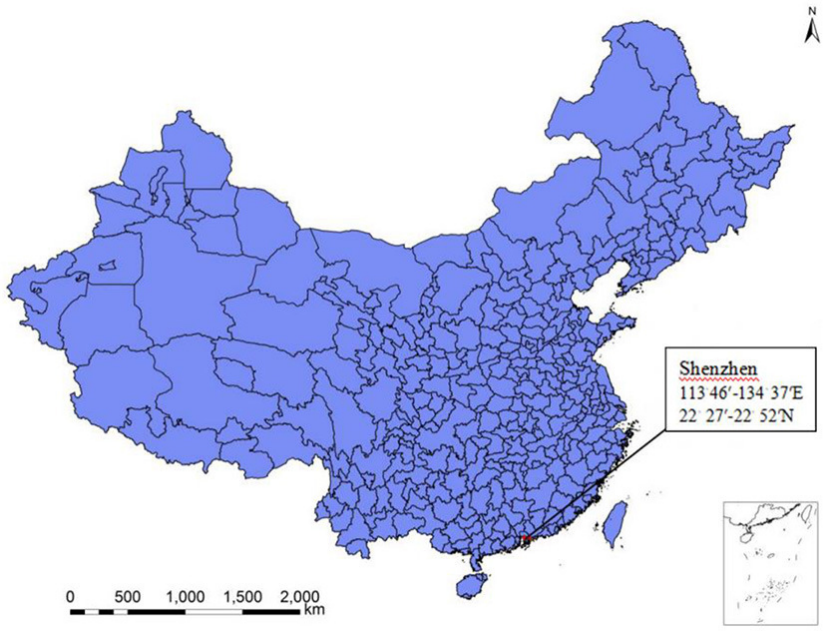
Geographical distribution of Shenzhen.

Shenzhen, one of the most developed cities in China (Figure 1), the government decided to build a “Shenzhen template” for “Healthy China 2030” Plan. In a document issued by the Shenzhen Municipal Government in 2020, it is stated that Shenzhen will strengthen the national health promotion work, regard health education as an important part of quality education in all education stages, and comprehensively improve the health literacy of its citizens. But at present, there is no research about the HL and HPL of community residents in Shenzhen. Therefore, we conducted an online survey to understand the status quo of HL and HPL among local residents in Shenzhen. The relationship status between HL and HPL is complicated and may not be simply summarized as “health literacy promotes health behavior” (28). We speculate that total HL should be positively correlated with HPL in general, but it may not always be positively correlated with HPL when it divided into different levels. When different total HL levels show other relationships with HPL, increasing total HL may not improve HPL, at this time, it may be necessary to analyze the effects of different HL types on HPL. Therefore, in this study, we grouped and classified HL into different groups and stages, and then discussed the relationship between different HL types and HPL to provide a basis for better public health policies improve the majority health.

## Methods

### Setting and participants

The survey was carried out in one of the ten sub-districts under the jurisdiction of the Baoan District, Shenzhen. Community residents belonging to this sub-district were the participants of this study. Shenzhen is a coastal city in Guangdong Province with a large permanent population (17,560,061), the population aged ≥15 is 14,906,680, accounting for 84.89%. According to the statistics report of Shenzhen city, Shenzhen is an economically developed and relatively young city with a large population in China (29). This study was a cross-sectional investigation examining the HL level and current situation of health lifestyles among Shenzhen community residents and analyzing the relationship between HL and HPL in different populations. We calculate the sample size according to the following sample size formula (30), with a 95% *CI* and, *P* = 0.05. Where n is the sample size, *N* = (14,906,680) is the population size, and *e* = (0.05) is the level of precision. Finally, we calculated the minimum sample size (400) for research.


$$\begin{array}{l} n=\frac{N}{1+N{\left(e\right)}^{2}} \end{array}$$

**Figure 2:**
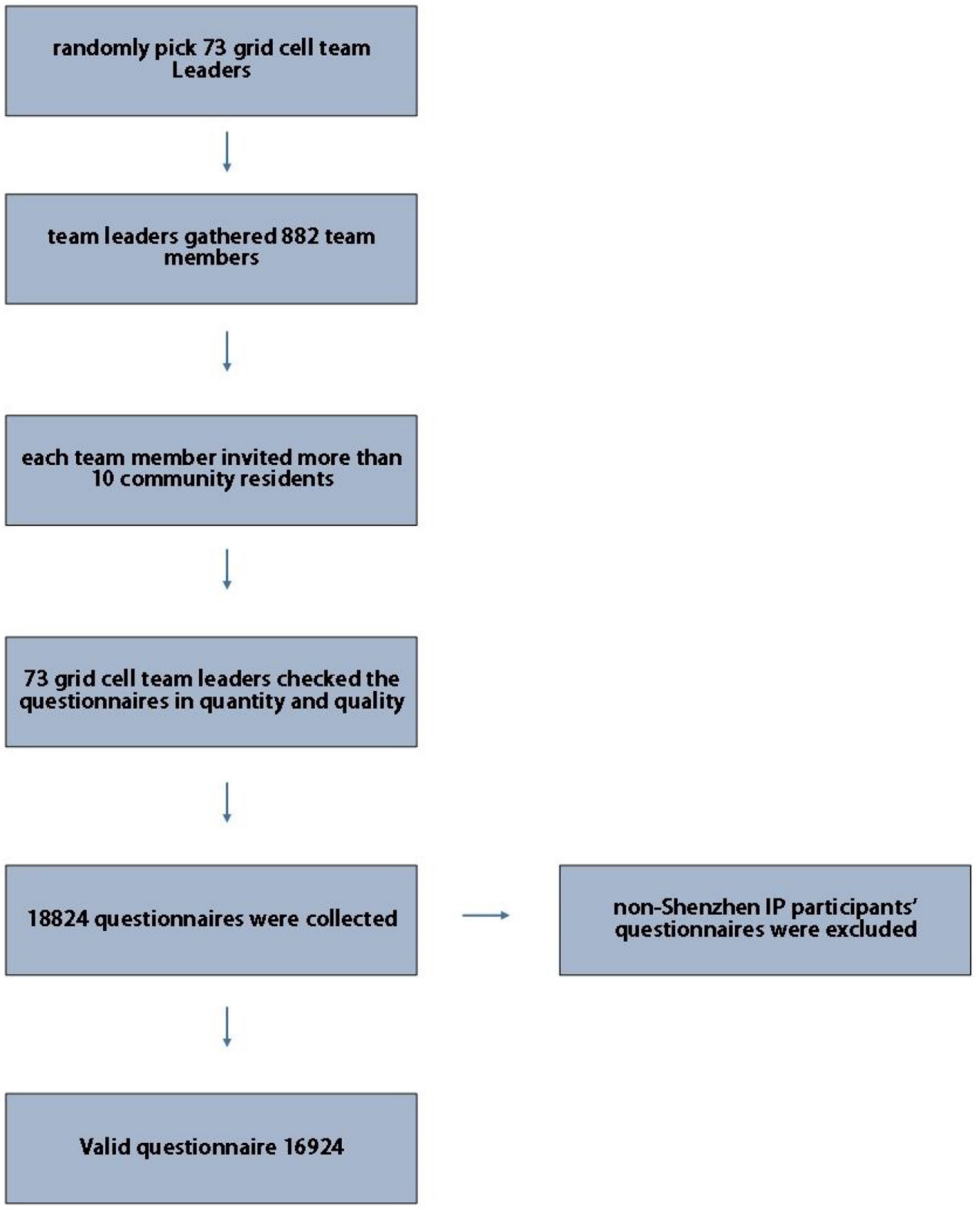
Questionnaire collection.

As part of this study, a “health network survey activity” was launched through the official WeChat account of a health supervision institute of Shenzhen. According to the document issued in 2007 (31), the Shenzhen municipal government has gridded the whole city. For this survey, 73 grid cells were randomly selected from the subordinate grids of the survey sub-district, and the snowball sampling method was used for data collection. In the first stage, 73 grid cell team leaders were gathered *via* a street office to answer the questionnaire. In the second stage, the team leader of each grid mobilized 882 team members to answer the questionnaire. In the third stage, each team member invited more than ten community residents to answer the questionnaire by sending a message on WeChat Moments or distributing the questionnaire link. We obtained each participant's consent before administrating the questionnaire. Each account could only fill out one questionnaire. In order to ensure that the survey is completed in quantity and quality, each team member will collect the list (name and mobile phone number) of 10 community residents who participated in the survey to the leader of the grid group. In order to ensure the scope of investigation, IP addresses in Shenzhen area are set up in the background of the system for screening. The liaison of the research group will check the completeness of the questionnaire in the background of the system in time, and feed back to the grid group for supplement and improvement if there are missing items. Residents click on the website, enter their name, mobile phone number, gender, date of birth and other information, then answer questions. There is no limit to the time for answering questions. To ensure the accuracy of the data in the process of data entry, double data entry and logical check are adopted. If any suspicious data is found, check the original data promptly. If it is clearly wrong and cannot be remedied, the data will be excluded. Figure 2 shows the questionnaire collection.

### Instruments

The two instruments used in this study were the Chinese Citizen Health Literacy Questionnaire and the Health-Promoting Lifestyle Profile II (HPLP-II).

#### Demographic information

Demographic information included gender, age, nationality, education level, occupation, family size, and personal annual income. Nationality was divided into Han and non-Han. Occupations included civil servants, teachers, medical personnel, public institution personnel, students, farmers, enterprise personnel, and others. Personal annual income was divided into four grades: < ¥50000 (<5), ¥50000– (5–), ¥100000– (10–), ≥¥150000 (≥15).

#### The Chinese citizen health literacy questionnaire

We used the Chinese Citizen Health Literacy Questionnaire to survey the health literacy of the residents, this questionnaire was developed by the China Health Education Center (32). The Chinese government used this questionnaire to survey the health literacy of Chinese residents (17), it has high reliability and good internal consistency in studies (25, 32, 33). This questionnaire has a Cronbach α coefficient of 0.939 in our study, indicating high reliability and good content validity. The questionnaire includes include 6 categories of HL: scientific health concept, infection diseases, chronic diseases, safety and first aid, medical care, health information. The questionnaire consists of 50 questions with a full score of 66: 1 point for each judgment question and single-choice question, and 2 points for each multiple-choice question. No points were given for either missing an answer or making a wrong choice. Achieving 80% of the full score of health literacy in the six categories was considered as having health literacy in this category. For each participant, if his total questionnaire score ≥80%, he will be judged to have basic health literacy.

#### Health-promoting lifestyle profile II

HPLP-II was invented by Walker et al. (34) in 1987 and used in this survey. The questionnaire contains 52 items and six dimensions of HPL: health responsibility (HR), stress management (SM), physical activity (PA), nutrition (N), interpersonal relation (IR), and spiritual growth (SG). The Cronbach α coefficient of this questionnaire was 0.981 in our study. The possible scores of this scale are 52–208, in which 52–90 points mean a failing score, 91–129 points are barely adequate, 130–169 points are good, and 170–208 points are excellent.

### Statistical analysis

HL was classified into different groups and stages. The demographic characteristics comparison between different levels of HPL (“failing and barely adequate” vs. “good and excellent”) in same HL stage was performed through the chi-squared test, and the *Grammer* value was calculated to assess the effect size for these comparisons. Partial correlation analysis was used to analyze the relationship between HL and HPL. Multiple linear regression analysis took demographic characteristics as the control variable to analyze the relationships between each type of health literacy and HPL in HL stage that HL correlated with HPL. Reward and punishment analysis was used to estimate the effect of each health literacy on HPL in HL stage when HL did not correlate with HPL and multiple linear regression analyzed the relationship between HPL and its six dimensions, standardized scores were used to compare different dimension scores.

A reward and punishment analysis (35) was used to estimate the effect of each health literacy problem on HPL. First, the total scores for each health literacy problem were divided into three levels: “fail,” “barely adequate,” and “excellent,” according to the proportion of 40, 40, and 20%, respectively. Next, we calculated the total proportion of “good” and “excellent” HPL in each type of health literacy level: “fail,” “barely adequate,” and “excellent.” The category of health literacy problems was determined based on three percentages:

Weak effect of literacy: Literacy has no obvious effect on HPL. There was no significant change in HPL regardless of the HL score.
Basic literacy: When the literacy performance is not good, the HPL decreases significantly, but when the literacy performance is good, the HPL does not increase significantly. When this kind of health literacy problem is raised from “fail” to “barely adequate,” the HPL will increase significantly, but when it is increased from “barely adequate” to “excellent,” the HPL does not increase significantly.
Performance literacy: The higher the literacy score, the better the HPL performance. When this kind of health literacy score is raised from “fail” to “barely adequate” and from “barely adequate” to “excellent,” HPL increases significantly.
Motivating literacy: When the literacy performance is not good, the HPL does not decrease significantly, but if the literacy performance is good, the HPL is significantly improved. When this kind of health literacy is promoted from “fail” to “barely adequate,” there is no significant increase in HPL, but a literacy improvement from “barely adequate” to “excellent” greatly improved HPL.

A two-tailed test and α-level of 0.05 were used in this study. The data analysis was conducted using SPSS version 25.0.

## Result

In this survey, a total of 18,824 questionnaires were distributed and 18,824 collected, of which 2,568 were unqualified and 16,921 effective, with an effective recovery rate of 89.89%, including 10,221 males (60.4%) and 6,700 females (39.6%). Specifically, as shown in Table 1, 52.5% of the participants were aged 25–35, 66% have a high school or higher education degree, family size of 76.4% participants were 3–5 persons. The HPL of residents in this survey were 624 (3.7%) “failing,” 6,126 (36.2%) “barely adequate,” 6,523 (38.5%) “good,” and 3,648 (21.6%) “excellent”.

**Table 1:** Basic demographic characteristics.

**Demographic characteristics**	**N**	**%**
**Gender**		
Male	10,221	60.4
Female	6,700	39.6
**Age (in years)**		
15–	4,203	24.8
25–	8,887	52.5
35–	3,901	18.3
45–	538	3.2
55–69	202	1.2
**Nationality**		
Han nationality	11,292	66.7
Non-Han nationality	5,629	33.3
**Education**		
Primary school	2,777	16.4
Junior high school	2,980	17.6
Senior high/vocational high/technical secondary school	5,188	30.7
Junior college and above	5,976	35.3
**Occupation**		
Civil servant	1,038	6.1
Teacher	1,454	8.6
Medical staff	2,767	16.4
Public institution personnel	5,193	30.7
Student	2,235	13.2
Farmer	861	5.1
Enterprise personnel	2,697	15.9
Other occupations	676	4.0
**Family size (persons)**		
1	133	0.8
2	751	4.4
3	4,858	28.7
4	3,966	23.4
5	4,109	24.3
6 and more	3,104	18.3
**Personal annual income (**¥**, ten thousand)**		
<5	2,477	14.6
5–	5,365	31.7
10–	6,340	37.5
≥15	2,739	16.2
Total	16,921	100

**Figure 3:**
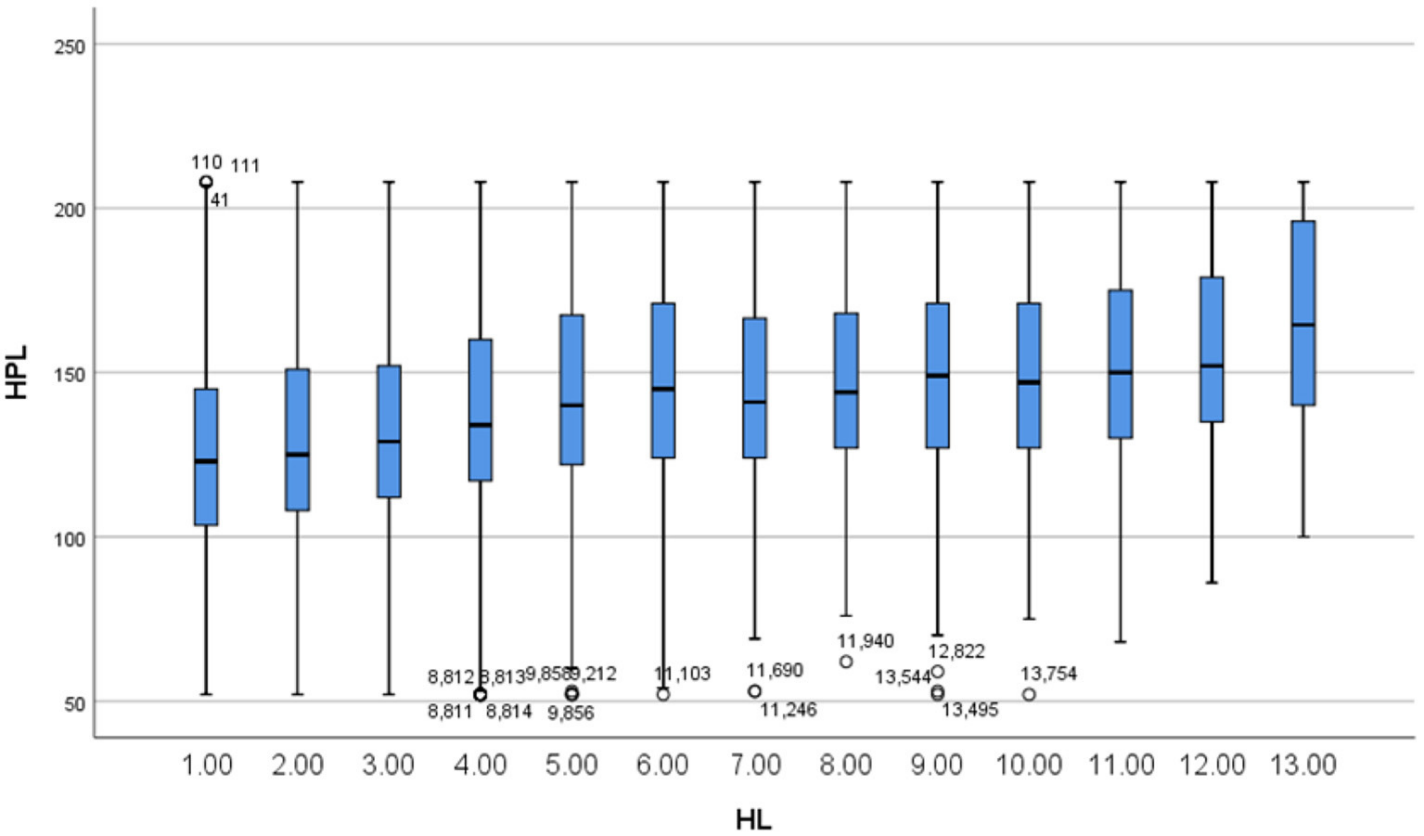
Boxplot of HL and HPL. HL: health literacy, the score range of health literacy was 2–66, and 5 points were divided into a group. HPL: health promoting lifestyle.

The survey results showed that the HP scores ranged from 2 to 66, and the HPL scores ranged from 52 to 208, the partial correlation coefficient of HL and HPL was 0.115 (*P* < 0.001). Figure 3 shows a boxplot of HL and HPL. In Figure 3, HPL increased in HL groups 1 to 5 and remained unchanged in groups 6 to 10, and the analysis of variance showed that HPL was different in groups 1 to 5 (*F* = 44.141, *P* < 0.001), but that there was no statistical difference among groups 6 to 10 (*F* = 2.018, *P* = 0.089), HPL significantly increased in groups 1 to 5 and remained stable in groups 6 to 10. Therefore, we temporarily classified groups 1 through 5 as one stage and named them the low-HL population, whereas groups 6 to 10 were classified as the medium-HL population. The total HL range of low-HL population was 2–26 with a total of 10,483 participants (61.95% of all participants); medium-HL had a score range of 27–51 with a total of 4,304 participants (25.44%); both populations were judged to lack basic health literacy (total HL ≤ 80%), which were the key to improving the public health.

We also analyzed the demographic characteristics of the different HPL levels in the low-and medium-HL populations (Table 2). The results show that there were differences in the distribution of demographic characteristics between different HPL levels, whether in low-HL or medium-HL populations. In the low-HL population, medium effect size values were obtained only for education and occupation differences between HPL levels; in the medium-HL population, medium effect size values were obtained only for occupation.

**Table 2:** Demographic characteristics analysis of low-and medium-HL populations.

**Low**						**Medium**				
**Basic demographic**	**failing and barely**	**Good and excellent**	***χ^2^***	***P***	**Grammer**	**Failing and barely**	**Good and excellent**	***χ^2^***	***P***	**Grammer**
**characteristics**	**adequate (%)**	**(%)**				**adequate (%)**	**(%)**			
**Gender**										
Male	3,620 (49.4)	3,703 (50.6)	28.103	*p <0.01*	0.05	633 (31.4)	1,380 (68.6)	5.413	*p <0.05*	0.04
Female	1,384 (43.8)	1,776 (56.2)				646(28.2)	1,645 (71.8)			
**Age (in years)**										
15–	1,469 (44.5)	1,834 (55.5)	23.5	*p* < 0.01	0.05	242 (31.3)	530 (68.7)	4.655	*p* > 0.05	0.03
25–	2,692 (49.3)	2,763 (50.7)				682 (29.7)	1615 (70.3)			
35–	665 (48.4)	710 (51.6)				279 (27.7)	727 (72.3)			
45–	97 (47.5)	107 (52.5)				67 (34.0)	130 (66.0)			
55–69	81 (55.5)	65 (45.5)				9 (28.1)	23 (71.9)			
**Nationality**										
Han nationality	2,331 (41.6)	3,279 (58.4)	184.966	*p* < 0.01	0.13	1,074 (29.0)	2631 (71.0)	7.768	*p* < 0.01	0.04
Non-Han nationality	2,673 (54.9)	2,200 (45.1)				205 (34.2)	394 (65.8)			
**Education**										
Primary school	1,476 (58.9)	1,029 (41.1)	366.887	*p* < 0.01	0.19	79 (35.9)	141 (64.1)	23.959	*p* < 0.01	0.07
Junior high school	1,270 (55.6)	1,014 (44.4)				205 (36.9)	350 (63.1)			
Senior high/vocational high/technical secondary school	1,366 (43.2)	1,797 (56.8)				429 (29.5)	1,023 (70.5)			
Junior college and above	892 (35.2)	1,639 (64.8)				566 (27.3)	1,511 (72.7)			
**Occupation**										
Civil servant	383 (49.2)	395 (50.8)	296.193	*p* < 0.01	0.17	36 (19.7)	147 (80.3)	49.193	*p* < 0.01	0.11
Teacher	612 (58.3)	437 (41.7)				70 (25.9)	200 (74.1)			
Medical staff	1,314 (59.2)	904 (40.8)				139 (36.5)	242 (63.5)			
Public institution personnel	1,463 (45.8)	1,730 (54.2)				396 (29.6)	943 (70.4)			
Student	631 (39.0)	986 (61.0)				136 (26.7)	373 (73.3)			
Farmer	181 (37.6)	300 (62.4)				74 (26.7)	203 (73.3)			
Enterprise personnel	323 (36.4)	564 (63.6)				306 (28.7)	760 (71.3)			
Other occupations	97 (37.3)	163 (62.7)				122 (43.7)	157 (56.3)			
**Family size (persons)**										
1–	64 (57.1)	48 (42.9)	39.916	*p* < 0.01	0.06	3 (27.3)	8 (72.7)	13.078	*p* < 0.05	0.06
2–	322 (55.9)	254 (44.1)				37 (35.2)	68 (64.8)			
3–	1,242 (46.8)	1,412 (53.2)				376 (27.8)	977 (72.2)			
4–	1,001 (44.8)	1,235 (55.2)				355 (29.4)	851 (70.6)			
5–	1,305 (46.7)	1,490 (53.3)				263 (28.5)	660 (71.5)			
6 and more	1,070 (50.7)	1,040 (49.3)				245 (34.7)	461 (65.3)			
**Personal annual income (**¥**, ten thousand)**										
<5	696 (48.9)	728 (51.1)	141.655	*p* < 0.01	0.12	296 (36.9)	507 (63.1)	39.1	*p* < 0.01	0.1
5–	1,669 (52.2)	1,529 (47.8)				452 (31.1)	1,001 (68.9)			
10–	2,112 (48.9)	2,208 (51.1)				370 (27.4)	981 (72.6)			
≥15	527 (34.2)	1014 (65.8)				161 (23.1)	536 (76.9)			

### Low-HL population

After controlling for demographic factors, the partial correlation analysis showed that in the low-HL population, the correlation coefficient between HL and HPL was 0.073 (*P* < 0.001), and except for the medical care health literacy (*r* = 0.019, *P* = 0.051), the correlation analysis *P*-values between the other five types of health literacy and HPL were <0.05. Because this model was an explanatory model, we still included “Medical care” in subsequent analysis. The multiple linear regression took demographic characteristics as the control variable to analyze the relationships between each type of health literacy and HPL in the low-HL population (Table 3). The first model, which included demographic characteristics, explained 8.7% of the variance of HPL (Model 1). The addition of health literacy to the second model (Model 2) accounted for an additional 2.3% of the variance. The results showed that health information literacy and chronic disease literacy could positively predict HPL in the low-HL population.

**Table 3:** Multiple linear regression analysis results for the low-HL population.

	**Model 1**			**Model 2**		
	**β**	***t***	***P***	**β**	***t***	***P***
Gender	0.036	3.765	<0.001			
Age (in years)	−0.049	−5.092	<0.001			
Education	0.161	15.706	<0.001			
**Occupation**						
Civil servant	−0.060	−3.351	0.001			
Teacher	−0.097	−4.787	<0.001			
Medical dtaff	−0.109	−4.144	<0.001			
Public institution personnel	−0.057	−1.964	0.050			
Student	0.003	0.149	0.881			
Farmer	0.034	2.223	0.026			
Enterprise personnel	−0.002	−0.107	0.951			
Other occupations	–	–	–			
Family size (persons)	−0.007	−0.750	0.453			
Personal annual income	0.121	12.425	<0.001			
Nationality	−0.077	−7.329	<0.001			
*R^2^* = 0.087, *F* = 77.196, *P* < 0.001						
Scientific health concept				0.004	0.371	0.71
Infection diseases				−0.072	−7.687	<0.001
Chronic diseases				0.080	7.792	<0.001
Safety and first aid				0.015	1.528	0.127
Medical care				−0.001	−0.152	0.879
Health information				0.080	7.995	<0.001
				*R^2^* = 0.110, *F* = 68.370, *P* < 0.001		
				*F_*change*_* = 45.026, *P_*change*_* < 0.001		

*β, Standardized Coefficients*.

### Medium HL population

#### Impact of each health literacy on HPL

The partial correlation analysis showed that the correlation coefficient between HL and HPL was 0.024 (*P* = 0.12). According to the calculation method introduced in statistical analysis, we calculated the total percentage of “good” and “excellent” HPL for each type of health literacy level: “fail,” “barely adequate,” and “excellent,” judging the influence of each type of health literacy on HPL. The reward and punishment analysis results showed that different health literacy problem have different influence on HPL, the scientific health concept, infectious disease, and safety and first aid literacy were weak-effect types of literacy, health information was a basic literacy, chronic disease was performance literacy, and medical care was a motivating literacy (Table 4).

**Table 4:** Reward and punishment analysis of each type of health literacy in the medium-HL population.

**Literacy problem**	**Fail (%)**	**Barely adequate (%)**	**Excellent (%)**	**Factor type**
Scientific health concept	69.881	70.180	70.784	Weak-effect
Infectious disease	72.740	69.679	70.588	Weak-effect
Chronic disease	59.028	69.134	76.384	Performance
Safety and first aid	68.935	71.948	67.113	Weak-effect
Medical care	68.848	70.134	83.871	Motivating
Health information	65.603	71.608	72.244	Basic

#### Relationship between HPL and its 6 dimensions

We analyzed the distribution between groups of a health-promoting lifestyle and its six dimensions in the medium-HL population (Table 5). The results of this data analysis showed that in the medium-HL population, there was no significant difference in the total HPL score, and the scores for the three dimensions—HR, SM and PA—were not significantly different, so we speculate that it may be HR, SM, and PA that cause HPL not to improve along with HL growth at medium-HL population.

**Table 5:** Analysis of the total health promotion living scale score and its six dimensions in the medium-HL population.

	**HR**	**SM**	**PA**	**N**	**IR**	**SG**	**Total score**
							**of HPL**
*F*	0.262	1.121	0.563	12.901	5.451	3.506	2.018
*P*	0.903	0.344	0.689	<0.001	<0.001	0.007	0.089

Standardized scores were used to compare the scores of each dimension in the medium-HL population, and the results were shown in Table 6. It shows that the HR and PA standardized scores (69.42 and 68.5) were lowest in HPL dimensions, while the SM standardized score (73.16) was highest. We then analyzed the relationship between HPL and its six dimensions in the medium-HL population with demographic factors as control variables. The analysis results are shown in Table 7. The tolerance and VIF were within the allowable range. The standardized coefficients of HR and PA were higher in the six dimensions, while the standardized coefficient of SM was the lowest in the six dimensions.

**Table 6:** Standardized score of each HPL dimension in the medium-HL population.

**Dimension**	**Total score of dimension**	**Score**	**Standardized score**	**Standardized score ranking**
		**$\bar{X}$ ±S**		
HR	36	24.99 ± 6.06	69.42	5
SM	32	23.41 ± 5.10	73.16	1
PA	32	21.92 ± 5.58	68.5	6
N	36	26.05 ± 5.50	72.36	4
IR	36	26.16 ± 5.34	72.67	3
SG	36	26.20 ± 5.80	72.78	2
Total score of HPL	208	148.73 ± 31.20		

*Standardized Score = (Average score of each dimension / Total score of each dimension) ×100*.

**Table 7:** Multiple linear regression analysis results for the medium-HL population.

	**Model 1**			**Model 2**		
	**β**	***t***	***P***	**β**	***P***	**Rank**
Gender	0.050	3.309	0.001			
Age (in years)	−0.017	−1.033	0.302			
Education	0.051	3.065	0.002			
**Occupation**						
Civil servant	0.123	6.427	<0.001			
Teacher	0.084	4.099	<0.001			
Medical staff	0.055	2.461	0.014			
Public institution personnel	0.146	4.827	<0.001			
Student	0.118	4.849	<0.001			
Farmer	0.120	5.837	<0.001			
Enterprise personnel	0.127	4.430	<0.001			
Other occupations	-	-	-			
Family size (persons)	−0.029	−1.898	0.058			
Personal annual income	0.120	7.525	<0.001			
Nationality	−0.050	−3.166	0.002			
*R^2^* = 0.039, *F* = 14.491, *P* < 0.001						
HR				0.193	<0.001	1
SM				0.164	<0.001	6
PA				0.179	<0.001	3
*N*				0.176	<0.001	4
IR				0.171	<0.001	5
SG				0.186	<0.001	2
				*R^2^* = 1, *F* = 29.32, *P* < 0.001		

*β, Standardized Coefficients*.

HR, SM and PA were not significantly different in medium-HL population; the effect of HR/PA on HPL were higher than SM (the standardized coefficients of HR and PA were 0.193 and 0.179, which were higher than SM), but the HR/PA standardized scores were lowest in the HPL dimensions, so it may be HR and PA that cause HPL unchanged between groups in the medium-HL population.

## Discussion

This study investigated the HL and HPL of Shenzhen community residents, the results showed that most Shenzhen community residents have insufficient HL, and HPL has potential to improve; HL is positively correlated with HPL on the whole, but HPL does not increase with the growth of HL in a part of population, so we divided total HL into different levels and analyzed the relationship between different HL types and HPL in different HL populations. Analysis showed that demographic characteristics were significantly different between different HPL levels in low-HL and medium-HL populations, medium effect size values for occupation was observed between different levels of HPL in the low-HL and medium- HL populations; in the low-HL population, health information literacy and chronic disease literacy can promote HPL; in medium-HL population, health information is a basic factor, chronic disease is performance factor, medical care is a motivating factor for HPL, and it is due to HR and PA that HPL cannot be improved in the medium-HL population.

Since its definition, several studies were carried out worldwide to estimate the HL levels of different populations and in different settings (36). HL is still low in many countries currently (12–17). Sufficient HL increases the individuals' ability to access, appraise, and use health-related information adequately, and to make good choices for their own health, while low levels of HL induce the inappropriate access and use of health resources (36). Lower HL is associated with more frequent use of emergency services, people with lower HL have less quality of life, a diminished life expectancy, worse life styles and are more likely to suffer from depression (15, 28, 37). In this study, the low-HL and medium-HL population accounted for 87.39% of the participants, and they lacked basic HL, which was apparently higher than the Chinese average level in 2021 (25.40%) (17). Lifestyle plays a vital role in explaining health and disease, poor lifestyle is already one of the top 10 causes of death in the United States (38) and is the pathogenic factor constituting 44.70% of the top 10 causes of diseases in China (21). Different demographic characteristics affected HPL in our study, especially occupational factors. 60.10% of the participants had good or excellent HPL, which is better than the survey results of Shandong Province, but Shenzhen residents still have great potentials for further progress to improve the population health.

HL positively correlated with HPL and HPL increased with the growth of HL in many studies (7–11, 22, 39, 40), but the relationship status between HL and HPL is complicated (28), these studies did not distinguish the effects of different HL stages/types to HPL. Some studies show different results. In a study of Taiwan adolescents found that adolescents with high and low HL did not significantly differ with some health promoting behaviors (41). The lack of a statistically significant relationship between HL and health promotion behaviors was also found in a study of urban black women in the United States (42). More importantly, in a study on training and counseling interventions provided by public health nurses during home visits, participants' health lifestyles did not improve when their HL increased (43). Our findings suggest that there may be different relationships between HL and HPL in the population, suggesting that different HL levels and types play different roles in HPL. Public health policy makers should pay attention to different relationship between different levels of HL and HPL, and it is advisable to develop different strategies for populations with different levels of HL.

### Limitations

This study has some limitations. The composition of the research sample does not allow to generalize our findings. In view of Shenzhen's huge population size and limited funds and manpower, we chose to randomly select some people as first participants, and then the first participants mobilized community residents to participate in the survey. Therefore, a selection bias should not be excluded. Randomized studies involving more representative samples would confirm our findings. Furthermore, this study is cross-sectional and requires more favorable support from subsequent studies.

## Conclusion

The influence of demographic characteristics on HL and HPL cannot be ignored, especially the influence of occupation. Health information literacy and chronic disease literacy are important in low-HL and medium-HL populations, medical care literacy can motivate people with medium-HL to adopt a healthy lifestyle, and medium-HL population need to increase HR and PA behaviors to improve HPL.

## Data availability statement

The raw data supporting the conclusions of this article will be made available by the authors, without undue reservation.

## Author contributions

JN, MY, and XM conceived and designed the study. LZ and JL performed the data collection and wrote the article. XP, DL, and LZ analyzed the data. JL, JZ, MS, XL, and XM were responsible for data analysis and interpretation. JN and MY revised and supplemented the manuscript. All authors read and approved the final manuscript.

## Funding

This work was supported by the grants from Major project of the school's high-level discipline construction project in 2021, under Grant 4SG21001G: Study on influencing factors, pathogenesis, accurate evaluation and comprehensive intervention of senile frailty based on community follow-up cohort and 2020 Provincial Education Reform Project: Mixed curriculum design and experiment of emergency response to public health emergencies 2020271.

## Conflict of interest

The authors declare that the research was conducted in the absence of any commercial or financial relationships that could be construed as a potential conflict of interest.

## Publisher's note

All claims expressed in this article are solely those of the authors and do not necessarily represent those of their affiliated organizations, or those of the publisher, the editors and the reviewers. Any product that may be evaluated in this article, or claim that may be made by its manufacturer, is not guaranteed or endorsed by the publisher.
